# Immune signatures of pathogenesis in the peritoneal compartment during early infection of sheep with *Fasciola hepatica*

**DOI:** 10.1038/s41598-017-03094-0

**Published:** 2017-06-05

**Authors:** Maria Teresa Ruiz-Campillo, Veronica Molina Hernandez, Alejandro Escamilla, Michael Stevenson, Jose Perez, Alvaro Martinez-Moreno, Sheila Donnelly, John P. Dalton, Krystyna Cwiklinski

**Affiliations:** 1 School of Veterinary Medicine, University of Cordoba, Cordoba, Spain; 2 School of Biological Sciences, Medical Biology Centre, Queen’s University of Belfast, Belfast, Northern Ireland UK; 3 The i3 Institute & School of Life Sciences, University of Technology, Sydney, Australia

## Abstract

Immune signatures of sheep acutely-infected with *Fasciola hepatica*, an important pathogen of livestock and humans were analysed within the peritoneal compartment to investigate early infection. Within the peritoneum, *F. hepatica* antibodies coincided with an intense innate and adaptive cellular immune response, with infiltrating leukocytes and a marked eosinophilia (49%). However, while cytokine qPCR analysis revealed IL-10, IL-12, IL-13, IL-23 and TGFβ were elevated, these were not statistically different at 18 days post-infection compared to uninfected animals indicating that the immune response is muted and not yet skewed to a Th2 type response that is associated with chronic disease. Proteomic analysis of the peritoneal fluid identified infection-related proteins, including several structural proteins derived from the liver extracellular matrix, connective tissue and epithelium, and proteins related to the immune system. Periostin and vascular cell adhesion protein 1 (VCAM-1), molecules that mediate leukocyte infiltration and are associated with inflammatory disorders involving marked eosinophilia (e.g. asthma), were particularly elevated in the peritoneum. Immuno-histochemical studies indicated that the source of periostin and VCAM-1 was the inflamed sheep liver tissue. This study has revealed previously unknown aspects of the immunology and pathogenesis associated with acute fascioliasis in the peritoneum and liver.

## Introduction

Fasciolosis is a disease of ruminants caused by the liver fluke *Fasciola hepatica*, and results in worldwide economic losses of greater than US $3 billion per annum^1–3^. It is also recognised by the World Health Organisation (WHO) as an important zoonosis; global infections are estimated between 2 and 17 million, with 180 million people at risk of infection^4–6^. The economic losses in ruminants are associated with the liver damage caused by parasites migrating through the definitive hosts resulting in poor food conversion, impaired fertility and reduced wool and milk production^7–11^.

Parasite migration begins following the ingestion of the infective encysted stage, the metacercariae, that excyst within the intestine as newly excysted juveniles (NEJs). The NEJs traverse the intestinal wall into the peritoneal cavity, where they continue to migrate through the liver capsule into the liver parenchyma. Those parasites that reach the liver, normally between four and six days post-infection, cause extensive tissue damage and haemorrhaging in the liver parenchyma resulting in the hepatic pathogenesis associated with acute fasciolosis^12^. *F. hepatica* secretes molecules that modulate the host immune response and induce the development of a Th2 response and concomitant inhibition of protective pro-inflammatory responses as the disease progresses to chronicity^12, 13^. This polarisation of the immune responses is sufficiently potent to influence the host’s susceptibility to co-infections with bacterial pathogens^13–19^.

Acute fasciolosis is especially problematic in sheep that die suddenly from haemorrhage and liver damage, particularly when large numbers of migrating immature flukes enter the liver; according to the National Animal Disease Information Services (NADIS) it is estimated that up to 10% of sheep at risk of infection in the UK will die of acute disease^20^. Clinical signs of infection include anaemia, dyspnoea, ascites and abdominal pain, which are also associated with sub-acute disease. Parasite populations resistant to the frontline anthelminthic used to treat acute fasciolosis, triclabendazole, are becoming more prevalent leaving farmers with no means of controlling acute infection^21^.

Studies have shown that as the parasite migrates through the intestinal epithelium clinical signs are not evident, although an immunological response is induced, as illustrated by the large number of immune cells infiltrating into the peritoneal cavity^22, 23^. Since the parasite migrates from the intestine to the liver via the peritoneum we considered that investigation of the peritoneal compartment of infected animals may provide new information of the early immune response in this compartment that can be exploited for vaccine development. At the same time, the data could also identify important host-specific proteins related to infection.

Proteomic analysis of the host response to *F. hepatica* has been carried out on host bile and serum^24, 25^, with the analysis of bile representing the chronic stages of infection when the adult parasites have migrated through the liver to the bile ducts^24^ and the serum representing the systemic response^25^. However, to date, the use of proteomics tools for the analysis of peritoneal fluid has only been reported in patients with uremia, endometriosis, ovarian cancer and following cases of peritoneal dialysis^26–29^, which has facilitated the development of biomarkers for these respective diseases/pathologies.

In the present study, we examined the changes that occur within the peritoneal compartment of sheep during the first 18 days of infection (dpi) with *F. hepatica*. This is the first differential proteomic analysis of peritoneal fluid comparing uninfected and infected sheep. Our data reveal immune signatures within the peritoneum, with the identification of molecular markers of significant parasite-induced liver pathogenesis. These molecular markers may be useful for vaccine development studies in the future, particularly in relation to defining correlates of protective immune responses.

## Results

### Early liver pathology caused by the migration of immature *Fasciola hepatica*

**Figure 1:**
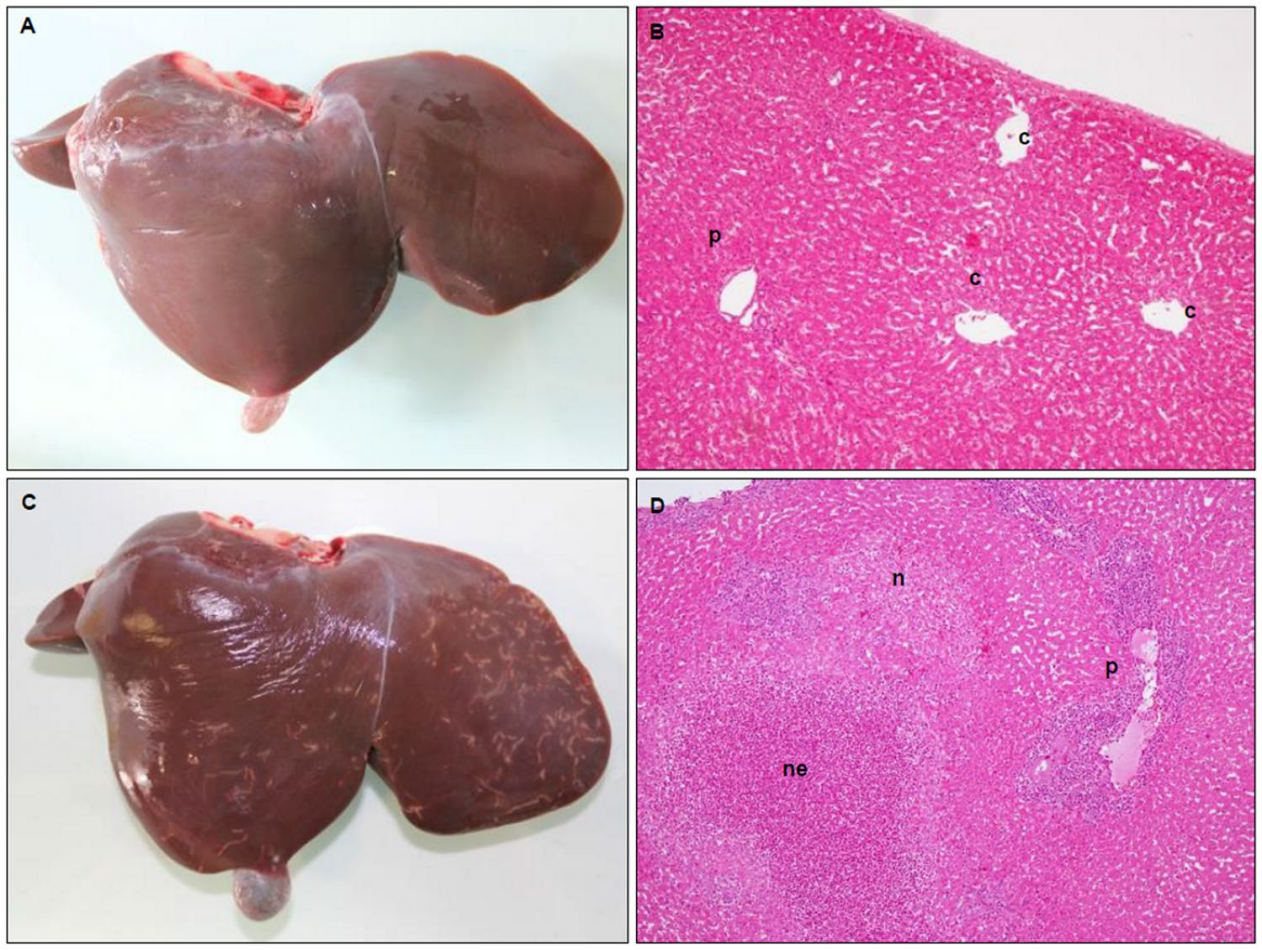
Comparison of gross and microscopical liver pathology between uninfected (**A** and **B**) and infected (**C** and **D**) animals. (**A**) Liver showing no apparent gross pathology. (**B**) HE stained liver microphotograph showing centrilubular veins (c) and portal spaces (p) with blood vessels and bile ducts and absence of inflammatory infiltrate (Magnification x400). (**C**) Liver showing white tortuous tracts caused by *F. hepatica*. (**D**) HE stained liver microphotograph displaying acute small necrotic foci without inflammatory infiltrate (n), acute necrotic foci with presence of abundant necrotic inflammatory cells (ne) and severe inflammatory infiltrate in adjacent portal spaces (Magnification x400).

No gross changes on either the diaphragmatic or visceral surface (Fig. 1A) were observed for the uninfected sheep. Liver from infected sheep (18 dpi) showed white/yellow foci and tortuous tracts ranging from 0.2–1.5 cm length located on the liver surface, mainly on the diaphragmatic aspect of the left lobe, consistent with *F. hepatica* infection (Fig. 1C). For some of the sheep, spots or small red tracts due to hyperaemia and haemorrhage could be observed.

At the microscopic level, histopathology revealed no hepatic damage in the uninfected sheep (Fig. 1B). In the infected animals, necrotic foci and necrotic tortuous tracts within the hepatic parenchyma, mainly involving sub-capsular areas were observed (Fig. 1D). Examination of the acute necrotic foci revealed a moderate inflammatory infiltrate, with a predominant infiltration of eosinophils and minimal infiltration of neutrophils (<1–2%), and peripheral focal haemorrhages. Portal spaces adjacent to necrotic foci showed severe inflammatory infiltrate suggesting that the route of the infiltrating inflammatory cells was through the portal vessels.

**Figure 2:**
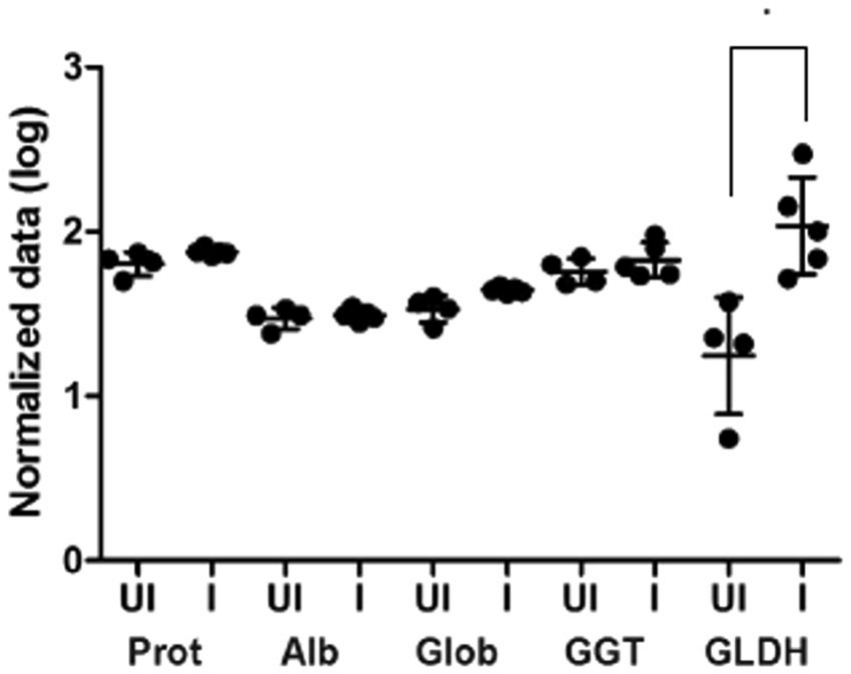
Liver enzyme profile. Serum from uninfected (UI) and infected (I) sheep, representing the mean (n = 5) of each group ± standard deviation were analysed for a variety of liver enzymes. The normalised data is shown by a dot plot on a log scale. Prot: total protein (g/l); Alb: albumin (g/l); Glob: globulin (g/l); GGT: gamma glutamyl-transferase (units/l) and GLDH: glutamate dehydrogenase (units/l). *P value = 0.01.

Liver enzyme analysis of the infected animals was consistent with the stage of liver fluke infection of the liver migrating parasites; elevated levels of glutamate dehydrogenase (GLDH) indicative of liver disease were observed, which were statistically significant when compared to the uninfected animals, based on normalised data (P value < 0.01). Furthermore, very low levels of gamma glutamyl-transferase (GGT), which is typically used as an indicator of chronic infection, were observed in both infected and non-infected groups (Fig. 2). Although the values observed for GLDH were significantly different between the groups, this was only observed when the data was normalised and large variation was observed between the animals suggesting that these serum enzymes may not be reliable markers for fasciolosis in sheep in the first 18 dpi. Analysis of liver enzymes within the peritoneal fluid found lower levels of GLDH compared to serum and no significant differences between the uninfected and infected animals (data not shown). GLDH originates from hepatocytes located in the centrilobular area of the liver that express high levels of this enzyme and directly release it into the circulation making it more readily detected in serum^30^.

### Immune signatures within the peritoneal compartment during acute *F. hepatica* infection

**Figure 3:**
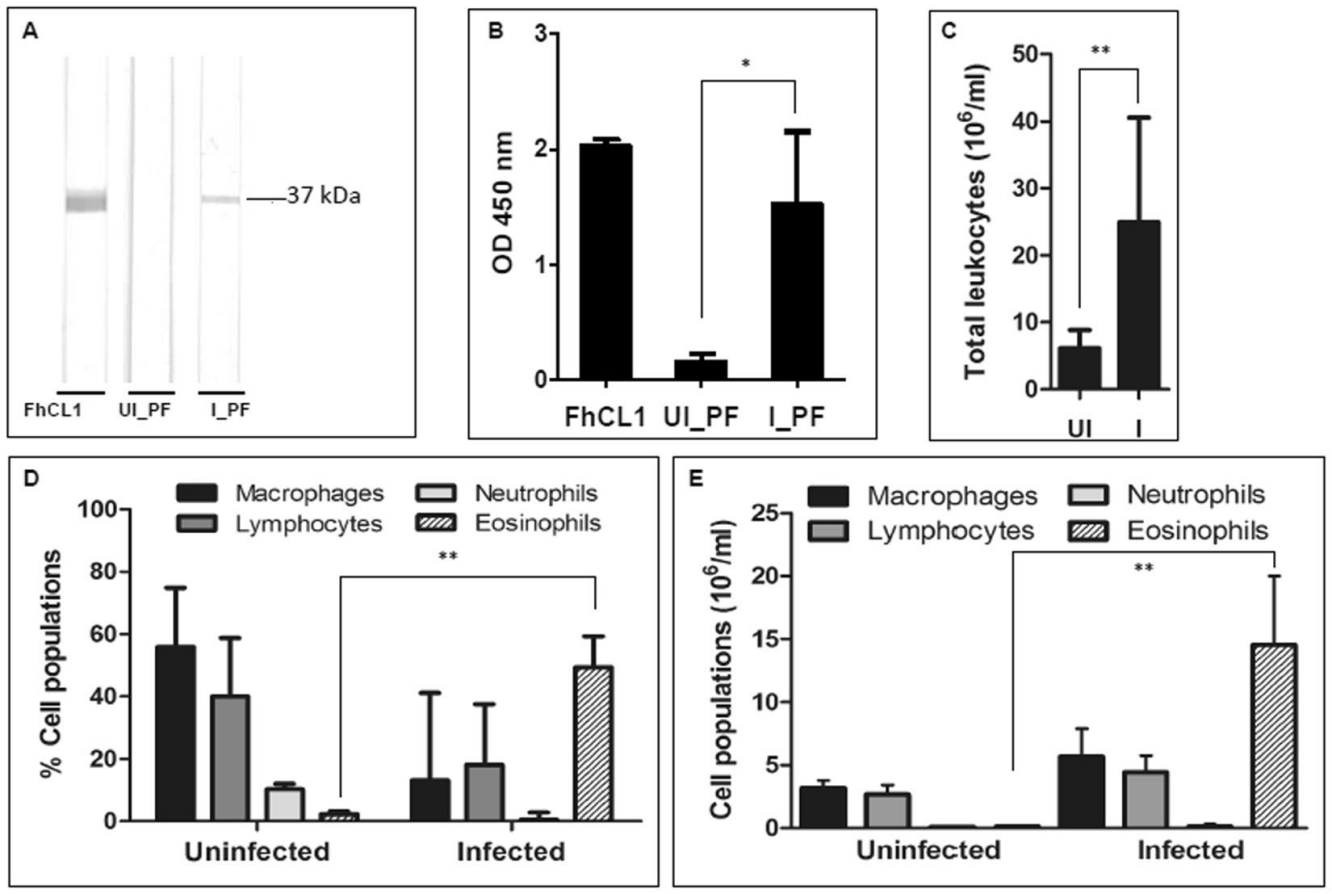
Peritoneal fluid humoral and cellular analysis. (**A**) Detection of *F. hepatica* cathepsin L1 (FhCL1) specific antibodies in peritoneal fluid by immunoblotting. Lane 1: FhCL1 positive control; Lane 2: peritoneal fluid from the uninfected pool (UI_PF); Lane 3: peritoneal fluid from the infected pool (I_PF). (**B**) IgG level response in peritoneal fluid against *F. hepatica* cathepsin L1 (FhCL1). 1: FhCL1 positive control; 2: uninfected (UI_PF); 3: infected (I_PF). (**C**) Total mean cell count per ml in the peritoneal fluid from the uninfected (UI) and infected (I) groups (n = 5; ± standard deviation is represented). (**D**) Mean differential cell count showing the percentages of macrophages, lymphocytes, neutrophils and eosinophils in the peritoneal fluid of uninfected and infected sheep (n = 5; ± standard deviation is represented). (**E**) Mean differential cell count of macrophages, lymphocytes, neutrophils and eosinophils in the peritoneal fluid of uninfected and infected sheep (n = 5; ± standard deviation) represented by cell number. *P value < 0.05; **P value < 0.01.

Using a combination of western blot and ELISA we evaluated the humoral immune response against *F. hepatica* in the peritoneal fluid of uninfected and infected animals (Fig. 3A,B). IgG antibodies against the recombinant *F. hepatica* antigen FhCL1 were markedly increased in the peritoneal fluid of infected sheep confirming *F. hepatica* infection (P value < 0.01). No FhCL1-specific antibodies were detected in uninfected sheep. This is the first report of the *F. hepatica* ELISA utilising FhCL1 being used for peritoneal fluid.

Analysis of the leukocyte profile found within the peritoneum revealed that the humoral response against *F. hepatica* coincides with a cellular immune response as observed by the statistically significant rise in total leukocyte number within the peritoneal fluid, from a mean value of 6 × 10^6^ ± 2.6 (SD) cells per ml in the uninfected animals to a mean value of 25 × 10^6^ ± 15.6 (SD) cells per ml (P value < 0.05; Fig. 3C). In addition, the populations of individual cell types were dramatically altered; most notably, with 49% of the cell population comprised of eosinophils in the infected sheep compared to 2% in the uninfected sheep, which represented a 94-fold increase in the actual number of eosinophils at 18 dpi compared to uninfected sheep (P value < 0.01). Whilst the proportion of macrophages and lymphocytes in the total cell counts were reduced following infection (Fig. 3D), due to the 4-fold increase in total cell count, the actual cell numbers increased 2-fold and 2.5-fold, respectively (Fig. 3E). There was also no evidence of mast cells or basophils within the cellular infiltrates, consistent with other studies in sheep^31^.

**Figure 4:**
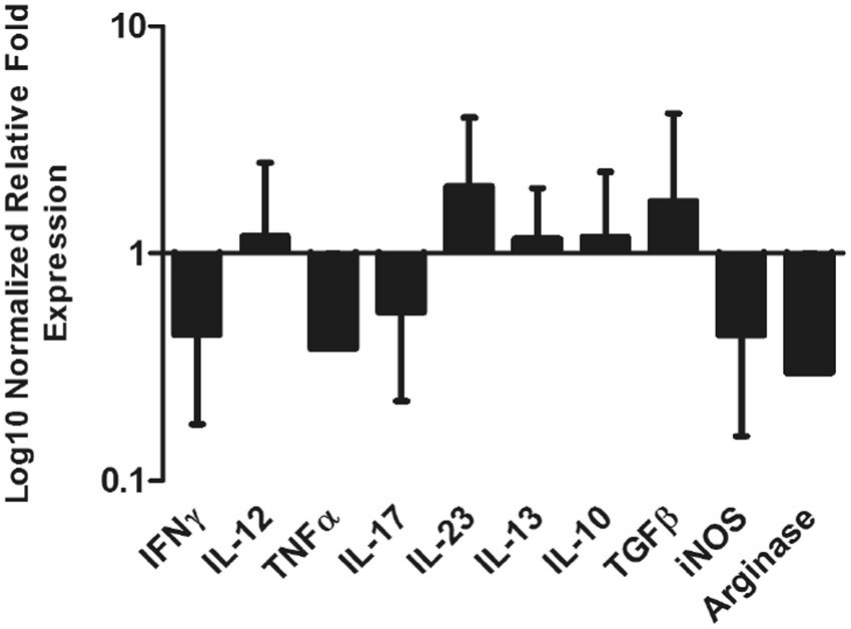
Analysis of relative quantitative cytokine gene expression within the cells of the peritoneal fluid, represented by normalized relative fold expression on log10 scale. Immune marker transcript abundance of the infected group was normalised against the abundance of the respective transcripts in the uninfected group. Transcript abundance of the uninfected group (not shown on graph) is equal to 1. Data represents mean + SEM of fold changes in target transcript abundance relative to three house-keeping genes: β-actin, B2M and GAPDH. Each bar represents data from five biological replicates; statistical analyses were performed using One Way ANOVA with Tukey’s post hoc tests, comparing the data from the uninfected with infected samples, which showed no statistical differences based on animal variation within each group.

An extensive panel of ovine cytokines related to parasite infection were selected for qPCR analysis of the transcripts isolated from total peritoneal cells. Some primers were obtained from the literature, while others were designed as part of this study using available ovine cytokine gene sequences (Table 1). Cytokines IL-1, IL-4 and IL-5, and the chemokine Eotaxin were not detected in our samples from infected and non-infected animals. Five cytokines (IL-10, IL-12, IL-13, IL-23 and TGF-β) were shown to be more highly transcribed by the peritoneal cells from infected animals compared to cells taken from uninfected animals, relative to three housekeeping genes, β-actin, beta-2-microglobulin (B2M) and glyceraldehyde 3-phosphate dehydrogenase (GAPDH) (Fig. 4). These results were not shown to be statistically significant due to the high variability between the animals.

**Table 1:** Primers for quantitative PCR (qPCR) cytokine analysis.

Cytokine	Forward sequence 5′-3′	Reverse sequence 5′-3′	Reference
IFN-γ	ATCTCTTTCGAGGCCGGAGA	ATTGCAGGCAGGAGAACCAT	110
IL-1	CACTGCCAGAAAATAAGCTGAAAC	TGATCAAGCAAATCGCCTGAT	111
IL-4	GGAGCTGCCTGTAGCAGACG	TTCTCAGTTGCGTTCTTTGGG	110
IL-5	CTGCTGATAGGTGATGGGAACTT	GGTGATTTGTATGCTGAGGAGTAGG	110
IL-10	CTGAGAACCATGGGCCTGAC	TCTCCCCCAGCGAGTTCAC	110
IL-12	GAATTCTCGGCAGGTGGAAG	GTGCTCCACGTGTCAGGGTA	110
IL-13	AGAACCAGAAGGTGCCGCT	GGTTGAGGCTCCACACCATG	110
IL-17	TGTGAGGGTCAACCTGAACAT	TGATAATCGGTGGGCCTTCTG	111
IL-23	GGGAAGTGGACAGAGGTTCC	CTGCCTCTCCAATCTGGGTG	112
TNF-α	CCCGTCTGGACTTGGATCCT	TGCTTTTGGTGCTCATGGTG	110
TGFβ1	GAACTGCTGTGTTCGTCAGC	GGTTGTGCTGGTTGTACAGG	113
Arginase	GCGGAAGTCAAGAAGACTGG	AGGTTGTCCATGCAAGTTCC	Current study±
iNOS	TAGAGGAACATCTGGCCAGG	TGGCAGGGTCCCCTCTGATG	Current study^
Eotaxin	ACAAGAAAATCTGTGTTGATCCCC	CCATGGCATTCTGGACCC	111
B2M	TTCTGTCCCACGCTGAGTTCA	CAACCCAAATGAGGCATCGT	*
Β-actin	ACCAGTTCGCCATGGATGA	AGCCGTTGTCAACCACGAG	110
GAPDH	GGTGATGCTGGTGCTGAGTA	TCATAAGTCCCTCCACGATG	111

*Pacheco, I.L., personal communication. ^±^Based on *Ovis aries* arginase 1 (XM_004011324). ^Based on the bovine sequences from Adler *et al*.^114^ using the *Ovis aries* nitric oxide synthase 2 gene (XM_004012488).

### ECM proteins associated with infection and liver pathology

**Figure 5:**
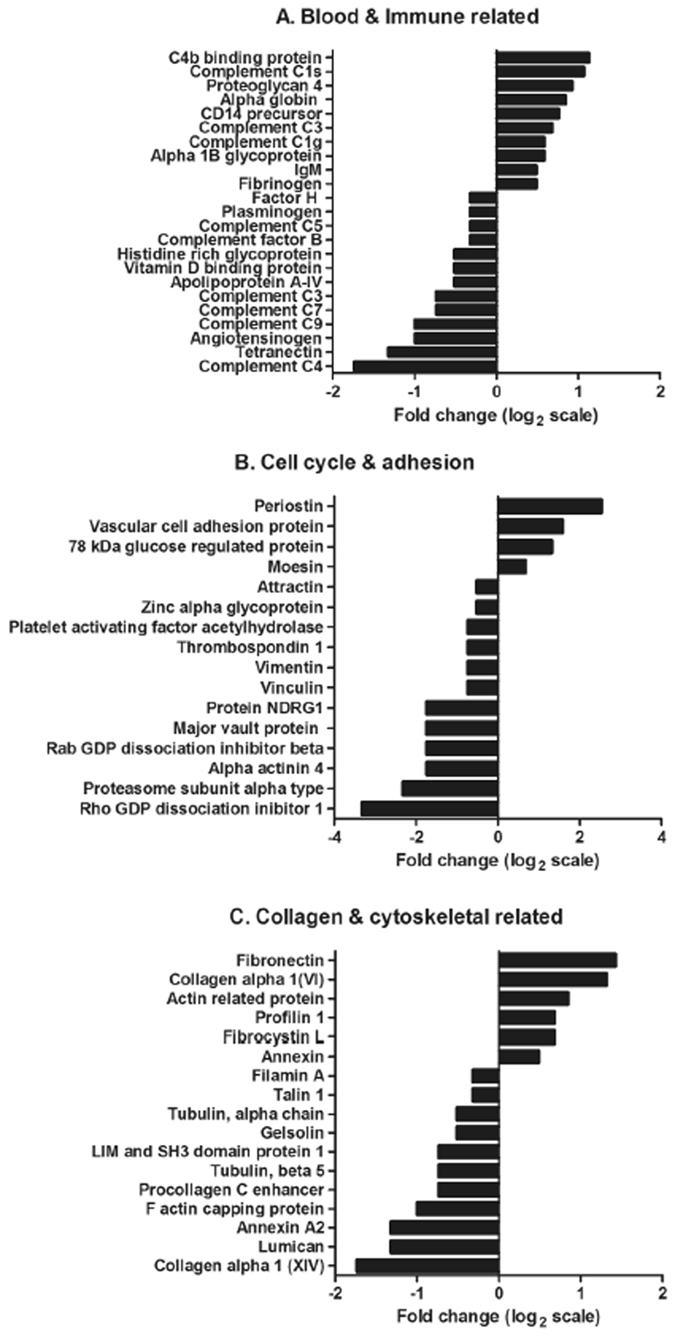
Expression profile of proteins identified within the peritoneal fluid relating to (**A**) blood, coagulation and the immune system, (**B**) cell cycle and cell adhesion, (**C**) collagen and cytoskeletal structure. The fold change, represented on a log 2 scale, was calculated based on the differences in protein concentration (emPAI values) between the uninfected and infected peritoneal fluid samples.

**Figure 6:**
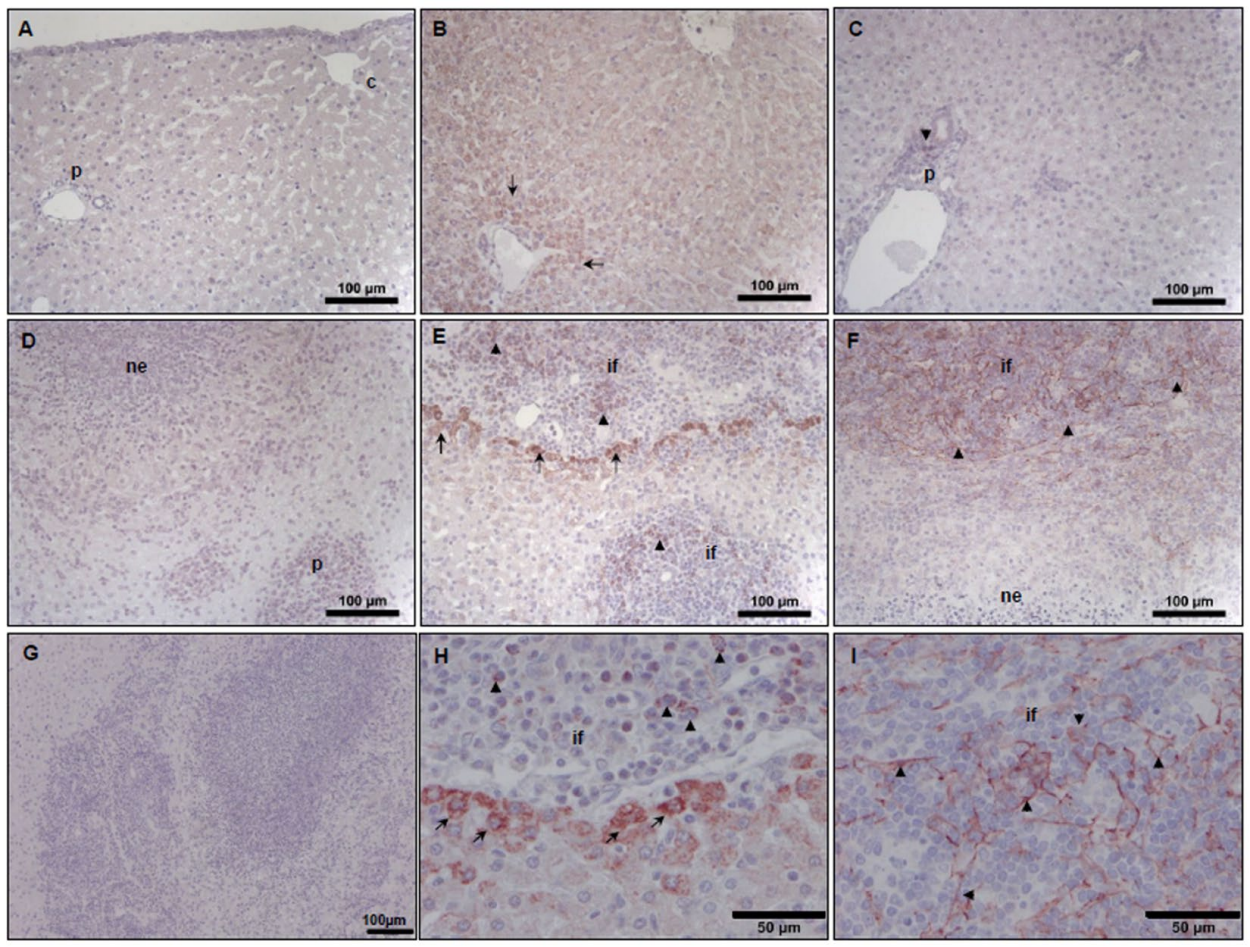
Immuno-labelling potential biomarkers of liver damage. Liver sections from uninfected (**A**,**B**,**C**) and infected sheep (**D**,**E**,**F**,**H**,**I**) were probed with periostin rabbit anti-mouse polyclonal antibody (**B**,**E** and **H**) and Vascular cell adhesion protein 1 (VCAM-1) rabbit anti-rat, mouse, human monoclonal antibody (**C**,**F** and **I**). Rabbit pre-immune serum (**A** and **D**) and staining using the goat anti-rabbit biotinylated secondary antibody only (**G**) were used as negative controls. Panels H and I are high powered images of panels E and F representing periostin and VCAM-1 staining, respectively. Periostin reactivity in the uninfected sheep (**B**) is found in the cytoplasm of hepatocytes, particularly in perilobular areas (arrows), whereas in the infected sheep (**E** and **H**) strong reactivity is found in hepatocytes (arrows) adjacent to inflammatory infiltrates (if) surrounding necrotic foci and in inflammatory cells (arrow heads). VCAM-1 is weakly expressed in the uninfected sheep (**C**) but strongly expressed in acute infection (**F** and **I**) particularly in the inflammatory infiltrates (if) showing a reticular pattern (arrow heads). ne: acute necrotic foci; if: inflammatory infiltrate; p: portal space. Scale bars represent 100 µm (**A**–**G**) and 50 µm (**H** and **I**).

Comparative analysis of the peritoneal fluid recovered from the uninfected and infected sheep identified a total of 176 proteins based on at least two unique peptides and the presence in both biological samples from experimental sheep trials carried out in Spain and the UK, which were classified according to putative function (Supplementary Table 1; Supplementary Fig. 1). Several structural proteins were identified related to the liver extracellular matrix (ECM), connective tissue and epithelium, including collagen VI structural unit proteins, fibronectin and fibrocystin, as well as proteins related to the immune system including immunoglobulins and components of the complement system (Fig. 5). Of particular note were the ECM-related proteins that showed the highest fold change differences in the infected animals when compared with the uninfected animals, namely, periostin (fold change 5.8) and vascular cell adhesion protein 1 (VCAM-1; fold change 3) (Fig. 5B). Periostin is associated with several inflammatory disorders and in particular, has been shown to be a systemic biomarker of airway eosinophilia in asthmatic patients being related with eosinophilic airway inflammation^32, 33^. VCAM-1, a leukocyte adhesion molecule expressed by cytokine-activated endothelial cells in culture, mediates mononuclear leukocyte infiltration in vessels and interstitium^34^. To confirm the presence of these proteins within the liver, we used an immunohistochemistry approach on sections of liver from the uninfected and infected sheep (Fig. 6). Periostin was found to be confined to the cytoplasm of hepatocytes and localised in the liver parenchyma in both the uninfected and infected animals (Fig. 6B,E and H, respectively). Interestingly, stronger periostin-related staining was observed in the hepatocytes surrounding the necrotic foci of infected animals. Furthermore, a moderate number of inflammatory cells were positive in the inner area of the acute necrotic foci and within the acute inflammatory infiltrate associated with the portal space (Fig. 6E and H). VCAM-1 was detected in the liver from infected animals, mostly in the inflammatory cells showing a reticular pattern; morphology compatible with dendritic cells within sites of acute inflammation. No VCAM-1 positive cells were found within the acute necrotic foci (Fig. 6F and I), compared with the very few positive cells with macrophage morphology localised in the portal spaces of the uninfected animals (Fig. 6C). Surprisingly, no VCAM-1 positive endothelial cells were detected in either the uninfected or infected animals.

## Discussion

The migration of *F. hepatica* NEJ from the intestine to the liver is a critical step in the parasites’ establishment of infection in the mammalian host. Entry of the liver is accompanied by the development of the parasite gut, metabolic alterations, rapid growth and a marked regulation of expression of >8000 genes^35^. From the hosts’ point of view, penetration and migration through the liver provokes severe pathogenesis as a result of intense inflammatory immune responses to fluke secretory antigens (including many proteases) and tissue damage. Prevention of tissue damage and inflammation is the primary aim of disease control; this makes the flukicide triclabendazole very attractive as a treatment since it is the only drug effective against the early migratory stages of the parasite. The spread of triclabendazole-resistant parasites, however, has left farmers without a means to control early and acute fasciolosis and consequently the disease has become increasingly prevalent in Europe and elsewhere^12, 21, 36^. Our focus is to develop an effective vaccine against *F. hepatica* that is directed at the early migratory stages to prevent their penetration into, and migration within, the liver. For this, we need to further understand the immune response of the host to these early invasive stages, particularly in the vicinity of their migration i.e. the peritoneal cavity.

The immunological profile in the peritoneum during early stages of fasciolosis reflects that specific immune signatures can be identified within the ovine peritoneal compartment. Analysis of the peritoneal fluid showed a clear antibody response characterised by the presence of anti-FhCL1 (a secreted protease) antibodies. Furthermore, there was intense cellular activity of both innate (macrophages, eosinophils) and adaptive (lymphocytes) immune cells. Most notable was the very marked eosinophilia (49% of immune cells). Eosinophilia is also observed within the peritoneum during the early stages of infection in rats^37^, and was observed by both histocytochemical studies^22, 23, 38^ and transcriptome analysis of *F. hepatica*-infected sheep liver^39, 40^. Marked eosinophilia is typical of helminth infections that drive Th2-dominant immune responses and in some cases, for example primary filarial infections^41^ and schistosomiasis^42, 43^, is associated with significant immune protection. However, the role of these cells in regulating immune responses to infection and their direct anti-parasite function is being heavily debated. Studies using eosinophil deficient mice infected with *Schistosoma mansoni* and *Nippostrongylus brasiliensis* have shown that the high levels of eosinophils may play a role in tissue remodelling rather than as agents of parasite damage (reviewed by^44^). Similarly Ohnmacht and colleagues^45^ suggest that the peritoneal cavity is a reservoir for eosinophils during helminth infection. Given the lack of resistance observed in sheep (reviewed by^36^) and our observations of marked eosinophilia within the peritoneum we would conclude they are not protective in primary responses in this species. Coupled with the lack of eosinophil degranulation proteins observed within the peritoneal fluid and the fact that the majority of the *F. hepatica* parasites at 18 dpi are present within the liver, this implies that the peritoneal cavity acts as a reservoir for non-activated eosinophils that disseminate from here to other compartments.

*F. hepatica* infection studies in mice^46, 47^ and ruminants^48–51^ show that the immune response is dominated by a Th2-type response. However, most studies in ruminants have examined infections at patent or chronic stages when significant parasite development and host tissue damaged has occurred^48, 49, 51^. Our present study is the first to investigate early immune responses in the peritoneum compartment at a very early stage of infection. Our cytokine analysis of the mixed population of peritoneal cells by qPCR detected increased transcription of cytokines IL-12 and IL-23 that are associated with pro-inflammatory processes. Increased gene transcription of TGF-β and IL-10 was also observed in the infected animals, with TGF-β being implied in anti-inflammatory responses inducing fibrosis and tissue regeneration. TGF-β and IL-10 also play a central role in minimizing pathology and enhancing tissue repair during helminth infections^52, 53^. Interestingly, we did not observe IL-4, but perhaps transcription of this cytokine is down-regulated by TGF-β as seen in *N. brasiliensis* infections in mice whereby Treg–derived TGF-β counterbalanced IL-4 expression^54^. Similarly, in *F. hepatica*-infected mice only low levels of IL-4 transcription can be detected at 14 days post infection^46^. Overall, our data imply that, the immune responses initiated within this mixed population of cells, comprised of 20% lymphocytes, prior to 18 days of *F. hepatica* infection in sheep is weak and not differentiated to a Th2 type typical of chronic infection. The potent skewing towards a Th2 response appears only to occur after the parasite has penetrated the liver parenchyma and caused significant tissue damage (12; Cwiklinski *et al*., unpublished).

To further describe the immuno-pathological responses to *F. hepatica* infection and complement previous studies of the peritoneal compartment^55–57^, we employed a novel approach to identify the proteins present within sheep peritoneal fluid during early *F. hepatica* infection using proteomics. This was performed by electrophoretically separating the proteins under denaturing conditions and then slicing fractions above and below the predominant host proteins (circa 67 kDa albumin; Supplementary Figure 1). While we likely missed proteins at similar migration to albumin we were able to identify 176 peritoneal fluid proteins. This data provides the first step to characterising the proteins that may play an important role in immunopathology and/or can act as biomarkers of infection. This study also reflects analysis of the host response following infection with different *F. hepatica* isolates, a UK isolate (South Gloucester) and an Italian isolate. Genetic analysis of these isolates has yet to be carried out, though recent studies have shown that *F. hepatica* is highly polymorphic and isolates within the UK show high levels of genetic diversity^35, 58^, indicating that these two isolates could exhibit differences. From the host’s point of view, comparative analysis of our proteomic data revealed a strong positive correlation between the biological replicates of each time-point (uninfected: r = 0.8799, P < 0.0001; infected: r = 0.7693, P < 0.0001), with the same proteins identified at comparable levels, based on emPAI values. As this study focussed on the early stages of infection, the number of parasites responsible for the infection cannot be quantified, so inferences of the virulence/pathogenicity of these isolates were not made as part of this study. However, based on similar pathology and immune responses across both experiments, these results indicate that despite potential genetic diversity, during the early stages of infection, the host response is comparable to each isolate.

The protein periostin is of particular interest because not only was it dramatically increased in the peritoneal fluid of infected animals it was also particularly abundant in the cytoplasm of hepatocytes surrounding the necrotic foci, within inflammatory cells of the acute necrotic foci and within the acute inflammatory infiltrate associated with the portal space. As several cell types express periostin, including epithelial cells, fibroblasts (peritoneal and liver) and hepatocytes^59–68^, the source of periostin within the peritoneal fluid may be damaged liver tissues. Alternatively, the protein could be secreted by the peritoneal eosinophils that predominate in the cavity. Periostin has been shown to be involved in cellular dedifferentiation, ECM deposition and angiogenesis^33, 63, 64^, and within the liver where it plays a role in wound repair and tissue remodelling it co-localises with and binds to the ECM proteins fibronectin, tenascin C and fibrillar collagen (I, II, V) to form a connective tissue structure^65^. As well as being involved in fibrosis, periostin could also play a role in promoting eosinophil recruitment and migration within the ECM as observed in asthmatic studies^32, 66–68^. It has been suggested that soluble periostin may drive adhesion of eosinophils to ECM fibronectin increasing their survival^68^. Interestingly, expression of periostin is strongly associated with Th2-mediated pathologies^69–71^ and has been proposed as a biomarker for Th2-driven/eosinophilic inflammation^66^. For example, elevated levels of periostin can be detected in the serum of patients with acute or chronic hepatitis^72^.

The abundance of the adhesion protein VCAM-1 within the infected peritoneal cavity was supported by immunohistochemistry analysis that showed its enhanced expression in liver tissue of infected sheep compared to non-infected animals. This is consistent with other studies showing an increase in VCAM-1 expression associated with chronic liver and biliary diseases^73^. As observed in this study, VCAM-1 expression is low in resting, undamaged cells but during liver inflammation its expression increases, and is believed to be important for recruitment of monocytes and lymphocytes^74–76^. Cannistra *et al*.^77^ showed that functional VCAM-1 is expressed on activated mesothelial cells and may play a role in the distal arm of leukocyte trafficking to the abdominal cavity. Intestinal epithelial cells are also capable of expressing VCAM-1 during mucosal inflammation^78^, which could be released into the peritoneum following migration of the NEJs across the intestine.

We identified several components of the complement system (C1-C9) within our proteomic data. In particular, C1 g and s subcomponents and C3, together with the C4b binding protein were shown to be increased at 18 dpi, with C3 being statistically upregulated (P value = 0.0029). C3 is the parent protein of C3a, which has shown to be an eosinophil chemo-attractant^79^ and therefore the proteolytic cleavage of this protein may be associated with the increase in peritoneal eosinophils we observed. The importance of the complement cascade system during *F. hepatica* infection is further highlighted by liver transcriptome analysis performed by Alvarez-Rojas and colleagues^39^ that reported the upregulation of genes related to the complement system two weeks post infection, particularly the C4b binding protein. *In vitro* studies have shown that complement activation on the surface of juvenile stages of *S. mansoni* can mediate parasite killing^80, 81^. While studies have shown that parasites, including *F. hepatica*, are able to inhibit both the classical and alternative complement pathways as part of their survival strategies^82, 83^, the possible interaction between the complement cascade and *F. hepatica* NEJs warrants further investigation.

Our proteomic analysis also detected components of the liver ECM within the peritoneal fluid that are likely associated with the liver damage caused by the digestive activity of the migrating parasites. Collagen VI was particularly abundant, perhaps not surprisingly as it is found throughout the connective tissue, forming branching networks that bind to other ECM proteins. This networking-forming collagen increases during liver fibrosis, and an increase of its soluble form in the circulation of patients with chronic liver disease is often seen as a marker for tissue damage and hepatic fibrosis^84–87^. Collagen VI gene expression was also found to be upregulated in 8-week post *F. hepatica* infection sheep livers by Alvarez-Rojas and colleagues^39^, indicating its role in liver re-modelling following parasite infection.

Another early marker of liver damage is the increased expression and deposition of the adhesion protein fibronectin^88–90^, which is actively secreted by hepatic stellate cells in response to liver injury^72^. We observed increased levels of fibronectin within the peritoneal fluid, which together with collagen VI, may be released from the liver as the NEJ actively migrates through the connective tissue within the liver, rather than penetrating through the fibrillary collagen structures (collagen I, III & V) at this early stage.

The ability to uncover host-parasite interactions can now be complemented through the availability of extensive bioinformatic datasets for both *F. hepatica* ^35, 91–93^ and various definitive hosts of this parasite, including the bovine and ovine genomes^94–96^. Our particular study was made possible through the availability of the most recent *Ovis aries* genome^96^. In addition, studies analysing host-specific responses using transcriptomics are now being carried out^40, 41, 97, 98^. A recent study by Alvarez-Rojas and colleagues^39^ exploring the livers of *F. hepatica* infected sheep highlighted the transcriptomic changes observed within the liver eight weeks post infection, including several upregulated genes linked to fibrosis (tgf-β related genes, calponin, transgelins and osteopontin) and markers of a T-cell response (BCL6, CD86, IL1R2, IL18BP, IL27RA, TGFβ and TNF). Osteopontin together with periostin, has been shown to mediate chronic rhinosinusitis inflammation by inducing a proliferative response of the ECM^99^. As fibrosis typically occurs four weeks post infection, periostin may be facilitating liver re-modelling without inducing fibrosis prior to the induction of other fibrosis-associated proteins, as has been observed in myocardial tissue^100, 101^. We observed no IFNγ expression at 18 dpi, which may be connected to IL18BP expression, a natural inhibitor of IFNγ^102^; a marker that requires further investigation. Although, the upregulation of TGFβ is consistent with our study, the remaining markers found to be upregulated at eight week post-infection are different, reflecting the different tissue/cells used for analysis and that the immune response profile changes during the course of infection.

Our study focussing at early infection in the peritoneum compartment has identified immune signatures associated with early fasciolosis. Given that we have shown here that serum levels of GLDH and GGT are not reliable markers of liver damage prior to 18 dpi, and that immune responses have not yet differentiated, this study has identified new molecules associated with early *F. hepatica* infection at this stage. These biological markers may be useful in future diagnostics and also in vaccine development studies to define correlates of protective immune responses.

## Methods

### Ethical statement

All animal work carried out in Spain was approved by the Bioethical Committee of the University of Cordoba (1118) and was carried out according to the Directive 2010/63/EU and Spanish (Ley 6/2013) directives for animal experimentation. Experimental procedures at the Agri-Food and Biosciences Institute (AFBI; UK) were carried out under license from the Department of Health, Social Services and Public by the Animal (Scientific Procedures) Act 1986 (License No. PPL 2771), after ethical review by the AFBI Animal Ethics Committee.

### Sheep infections

Trial one: Ten 8-month-old male Merino-breed sheep (Spain) were allocated in two groups; uninfected (n = 5) and infected (n = 5). The infected group was orally infected with 150 *F. hepatica* metacercariae (South Gloucester isolate; Ridgeway Research Ltd) administered in gelatine capsules with a dosing gun. Animals were killed at 18 days post-infection (dpi) by intravenous injection of thiobarbital. At necropsy, the visceral and diaphragmatic aspects of the liver were photographed for gross evaluation. Tissue samples from the left and right hepatic lobes were fixed in 10% neutral buffered formalin and embedded in paraffin wax. For histopathology, 4 µm paraffin wax liver sections were stained with haematoxylin and eosin (HE).

Trial two: Ten 6 month-old male Dorset cross sheep (UK) were allocated in two groups; uninfected group (n = 5) and infected group (n = 5). The infected group was orally infected with 150 *F. hepatica* metacercariae (Italian isolate: Ridgeway Research Ltd) administered in water. Animals were euthanised at 18 dpi by captive bolt. At necropsy, the visceral and diaphragmatic aspects of the liver were photographed for gross evaluation.

### Recovery of peritoneal fluid

Peritoneal washing was conducted as previously described by Zafra *et al*.^22, 23^. The recovered peritoneal fluid was centrifuged at 430 × g for 5 min and the supernatant and cell pellet retained. The cell pellet was re-suspended in 1 ml DPBS and erythrocyte contamination removed using an erythrolysis buffer (155 mM Ammonium chloride, 10 mM Potassium bicarbonate, 0.1 mM EDTA). Cell viability and total cell count was assayed by trypan blue staining. Cell smears stained using panoptic stain for the differential cell counts were also performed using Vectabond-treated slides to determine the percentage of macrophages, lymphocytes, neutrophils and eosinophils within the cell pellet. This was calculated as a percentage of 100 cells, using differential nucleus and cytoplasm colour granule morphology to determine cell type.

The peritoneal fluid supernatant was centrifuged as follows, discarding any recovered pellet at each step: (1) 1720 × g for 10 min; (2) 25,000 × g for 30 min. Samples of the peritoneal fluid supernatants was concentrated (15-fold) by centrifugation in an Amicon Ultra-15 centrifugal filter unit (4000 × g at 4 °C), followed by a final centrifugation at 21,000 × g for 20 min at 4 °C. Equal protein concentrations (20 µg) from the concentrated peritoneal fluid supernatants were taken from the five animals of both the uninfected and infected groups, which were pooled for proteomic analysis.

### Detection of anti-*Fasciola hepatica* cathepsin L1 (rFhCL1) antibodies in the peritoneal fluid by ELISA

Flat-bottom 96 well microtitre plates (Nunc MaxiSorp) were coated with 0.5 μg/ml of FhCL1 antigen and incubated overnight at 4 °C. After 3 washes with PBS 0.05% Tween 20 (PBST; pH 7.4), 200 μl/well of blocking buffer (5% skimmed-milk powder diluted in PBST) was added and incubated for 1 h at room temperature (RT). After washing 3 times, 100 μl of the rabbit anti-FhCL1^103^ and concentrated peritoneal fluid samples diluted 1:6400 in PBST were added to the microtitre plates in triplicate and incubated for 1 h at 37 °C. After washing 4 times, the plate was incubated with 100 μl/well of 1:10,000 polyclonal donkey anti-sheep IgG conjugated to horseradish peroxidase (Novex) for 1 h at 37 °C. After washing 5 times, 100 μl TMB substrate (3,3′,5,5′-Tetramethylbenzidine Liquid Substrate Supersensitive) was added to each well. Following 15 min incubation the reaction was stopped with the addition of 100 μl 2 M sulphuric acid. A peritoneal fluid sample was considered to be positive when the OD-value, determined at a wavelength of 450 nm, was greater than the OD mean of peritoneal fluid samples from the uninfected group plus 2 standard deviations.

### Detection of *Fasciola hepatica* cathepsin L1 (rFhCL1) specific antibodies in peritoneal fluid by immunoblotting

SDS-PAGE was performed using 4–12% NuPAGE Bis-Tris gels and 5 μg of recombinant FhCL1 in 50 µl of NuPAGE LDS sample buffer, followed by protein transfer to a nitrocellulose membrane (0.2 μm pore size). The nitrocellulose membrane was incubated in blocking buffer (5% skimmed-milk powder in TBST; 20 mM Tris-HCl, 150 mM NaCl and 1% Tween20, pH 7.0) for 1 h at RT. The membranes were probed overnight at RT with a pool of concentrated peritoneal fluid from the uninfected and infected groups diluted 1:1000 in TBST or as a positive control an anti-FhCL1 antibody^103^ diluted 1:1000 in TBST. Following 3 × 10 min washes in TBST, the membranes were probed with an alkaline phosphatase conjugated polyclonal donkey anti-sheep IgG antibody (Sigma) diluted 1:5000 for 1 h at RT, followed by 3 × 10 min washes in TBST. Immuno-reactive bands were visualised using NBT/BCIP (Sigma).

### Serum levels of liver enzymes

Analysis of liver enzymes within the plasma of all animals was carried out for the following parameters: total protein, albumin, globulin, gamma glutamyl-transferase (GGT) and glutamate dehydrogenase (GLDH).

### Quantification of the cytokine expression in peritoneal cells by quantitative PCR (qPCR)

Messenger RNA (mRNA) was extracted from the peritoneal cells (3 × 10^6^ cells) using QIAzol (Qiagen) followed by DNase treatment. First strand cDNA was synthesised using Superscript™ II RNase H-Reverse Transcriptase and random primers (Life Technologies). qPCR reactions were performed in 20 µl reaction volume in triplicate, using 2 µl cDNA diluted 1:20, 10 µl of Platinum^®^ SYBR^®^ Green qPCR SuperMix-UDG kit (Life Technologies) and 1 µM of each primer (Table 1). qPCR was performed using the following cycling conditions: 95 °C: 10 min; 39 cycles: 95 °C: 10 s, 55 °C: 15 s, 72 °C: 20 s; 72 °C: 5 min. Relative expression analysis was performed manually using Pfaffl’s Augmented ΔΔCt method^104^ whereby the comparative cycle threshold (Ct) values of the samples of interest are compared to a control and normalised to three housekeeping genes, β-actin, B2M and GAPDH, according to a modified tool from geNorm. In order for this method to be valid, amplification efficiencies of individual reactions were verified using the comparative quantification package within the Rotor-Gene Q software v2.1.0. Annealing temperatures and melt-curve analysis was also carried out to check for single DNA products produced by these primer sets.

### Mass spectrometry analysis of ovine peritoneal fluid

Pooled peritoneal fluid supernatant samples from the uninfected and infected groups from the two experimental trials were precipitated using trichloroacetic acid (TCA), followed by analysis by 1-DE using a 4–12% SDS PAGE (Criterion XT Bis-Tris; BioRad). In-gel trypsin digestion was carried out followed by ES/MS-MS analysis using an Ekspert NanoLC425 (Eksigent) coupled to a 5600+ mass spectrometer (AB Sciex) equipped with a nanoelectrospray ion source. Peak list files were generated by Paragon and Progroup algorithms (Protein Pilot version 5.0; Sciex) using default parameters.

### Database searching and criteria for protein identification

All MS/MS spectra were analysed with Mascot (version 2.4.0), against the Uniprot sheep protein databank (27174 entries), assuming digestion with trypsin with 2 missed cleavages permitted. Fragment and parent ion mass tolerance for Mascot were set at 0.10 Da. Carbamidomethylation of cysteine was specified as a fixed modification and oxidation of methionine was specified as a variable modifications. Scaffold (version 4.6.2; Proteome Software Inc) was used to validate MS/MS based peptide and protein identifications. Peptide identifications were accepted if they could be established at greater than 95% probability to achieve an FDR less than 1% by the Scaffold Local FDR algorithm^105^. Protein identifications were accepted if they could be established at greater than 95% probability to achieve an FDR less than 1% and contained at least 2 identified peptides. Protein probabilities were assigned by the Protein Prophet algorithm^106^. Proteins that contained similar peptides and could not be differentiated based on MS/MS analysis alone were grouped to satisfy the principles of parsimony.

Protein abundance was calculated by Scaffold software based upon the normalised exponentially modified protein abundance index (emPAI) protocol. A normalization minimum value of 0.05 was added to all values to compensate for null or zero values and to allow for log transformation of the data. The averages of the emPAI values from the biological replicates were used for statistical analysis of protein abundance, resulting in an overall fold change difference being calculated relative to the uninfected group. Multiple pairwise t-tests were performed in Scaffold; P value < 0.05 was deemed statistically significant.

### Liver Immunohistochemistry (IHC)

Three µm paraffin wax liver sections were analysed using the avidin-biotin-peroxidase complex (ABC) method. Tissue sections were dewaxed, rehydrated and endogenous peroxidase activity was exhausted by incubation with 0.3% hydrogen peroxide in methanol for 30 min at RT. Two different antigen retrieval pre-treatments were used^107, 108^: (1) Detection of periostin: 0.01 M sodium citrate buffer, pH 6, heated for 20 min; (2) Detection of VCAM-1: Tris-EDTA buffer, pH 9, heated for 30 min. Sections were washed in PBS (pH 7.2) and incubated with 20% normal goat serum (ImmunoPure) for 30 min at RT. Endogenous liver biotin was blocked using the Avidin/Biotin blocking kit (Vector Laboratories). Overnight incubations at 4 °C were carried out using the following primary antibodies, rabbit anti-human periostin polyclonal antibody (LifeSpan BioSciences) and VCAM-1 rabbit anti-mouse monoclonal antibody (also cross reacts with rat & human; Abcam) both diluted 1:500 in PBS containing 10% normal goat serum. Following washing in PBS, the sections were incubated with the secondary antibody (goat anti-rabbit biotinylated antibody; Dako) diluted 1:200 in PBS containing 10% normal goat serum for 30 min at RT. After washing in PBS, the sections were incubated with the ABC complex (Vectastain ABC Elite Kit) for 1 h at RT in darkness, washed in 0.05 M Tris buffered saline (pH 7.6) and then incubated in the chromogen solution (Vector NovaRED Peroxidase Substrate Kit). Finally, the sections were counterstained with Harris’ hematoxylin and mounted in Eukitt quick-hardening mounting medium (Sigma).

### Statistical Analysis

Mann Whitney *U* tests and One Way ANOVA with Tukey’s post hoc tests performed on GraphPad Prism version 6.00 were used for statistical comparisons. P values of <0.05 was considered to be statistically significant. Correlation analysis of the proteomic data was performed on GraphPad Prism version 6.00.

### Accession Codes

The mass spectrometry proteomics data have been deposited to the ProteomeXchange Consortium via the PRIDE^109^ partner repository with the dataset identifier PXD005548 and 10.6019/PXD005548.

## Electronic supplementary material

Supplementary Information
