# Supplementary material for: Immune signatures of pathogenesis in the peritoneal compartment during early infection of sheep with Fasciola hepatica
Source: Sci Rep. 2017 Jun 5;7:2782. doi: 10.1038/s41598-017-03094-0 (PMC5459796; doi:10.1038/s41598-017-03094-0)

Immune signatures of pathogenesis in the peritoneal compartment during early infection of sheep with Fasciola hepatica

Maria Teresa Ruiz[1], Veronica Molina Hernandez[2], Alejandro Escamilla[1], Michael Stevenson[2], Jose Perez[1], Alvaro Martinez-Moreno[1], Sheila Donnelly[3], John P. Dalton[2], Krystyna Cwiklinski[2]

Supplementary Information

Supplementary Table 1: Proteins identified in the peritoneal fluid of uninfected and *Fasciola hepatica* infected sheep.

Supplementary Figure 1: Proteomic analysis of peritoneal fluid. (A) SDS-PAGE carried out on pooled samples of peritoneal fluid from the uninfected (UI) and infected (I) groups, represented by 4-12% SDS-PAGE gels stained with SYPRO ruby. * Position of albumin. Arrows indicate the section of gel used for proteomics analysis. (B) Proteins identified within the peritoneal fluid that show upregulation in the infected animals based on protein concentration. The fold change, represented on a log 2 scale, was calculated based on the differences in protein concentration (emPAI values) between the uninfected and infected peritoneal fluid samples. (C) Volcano plot analysis of statistically significant differences between the uninfected and infected animals, depicted in green (P value <0.05).

Supplementary Table 1: Proteins identified in the peritoneal fluid of uninfected and *Fasciola hepatica* infected sheep

					emPAI value				Unique peptide				Protein Identification Probability			
#	Accession Number	Annotation	T-Test (p-value): *(p < 0.05)	Fold Change by Category	UI PF	UI PF2	I PF	I PF2	UI PF	UI PF2	I PF	I PF2	UI PF	UI PF2	I PF	I PF2
1	W5NX51_SHEEP	Apolipoprotein A-I preproprotein	0.44	1.2	344.54	216.36	359.19	328.1	36	31	31	33	100%	100%	100%	100%
2	Q7M371_SHEEP	Plasma proteinase inhibitor	0.84	0.8	114.64	344.37	313	66.634	3	3	3	3	100%	100%	100%	100%
3	Q1KYZ8_OVIMU	Beta globin chain	0.9	0.9	9.3193	13.396	2.0801	18.3	9	11	6	9	100%	100%	100%	100%
4	I1WXR3_SHEEP	Alpha-1-antitrypsin transcript variant 1	0.87	1.1	12.617	10.457	7.359	17.636	18	16	16	13	100%	100%	100%	100%
5	ACTB_SHEEP	Actin	0.79	1.2	5.922	10.434	4.3895	15.588	16	19	18	20	100%	100%	100%	100%
6	W5NWM2_SHEEP	Apolipoprotein A-IV	0.44	0.7	10.773	16.889	4.9407	13.078	23	23	20	19	100%	100%	100%	100%
7	C6ZP47_OVIMU	Alpha globin	0.58	1.8	3.7588	4.4753	2.2932	12.72	5	6	4	6	100%	100%	100%	100%
8	W5PSQ7_SHEEP	Immunoglobulin lambda light chain	0.5	0.8	12.95	10.028	6.6861	11.63	9	8	9	9	100%	100%	100%	100%
9	W5NRI1_SHEEP	Complement C3	*0.0029	1.6	6.6384	6.6764	10.851	10.423	28	24	31	27	100%	100%	100%	100%
10	W5QHZ5_SHEEP	Ig kappa chain	0.92	1.1	22.26	3.9946	18.239	10.204	8	6	8	6	100%	100%	100%	100%
11	TTHY_SHEEP	Transthyretin	0.68	1.3	5.9848	4.1595	3.7807	9.0602	6	7	5	7	100%	100%	100%	100%
12	ANT3_SHEEP	Antithrombin-III	0.99	1	8.8453	3.5971	4.5448	7.8352	21	19	18	18	100%	100%	100%	100%
13	W5P6Q5_SHEEP	Profilin 1	0.58	1.6	2.4621	3.3737	2.0174	7.3486	4	4	4	4	100%	100%	100%	100%
14	W5Q5H8_SHEEP	Fibrinogen alpha chain	0.64	1.4	2.6886	4.2678	2.5888	6.8859	23	23	26	27	100%	100%	100%	100%
15	W5NSA6_SHEEP	Alpha-2-macroglobulin	0.99	1	6.6939	4.0887	4.242	6.5783	58	43	55	46	100%	100%	100%	100%
16	W5Q5A6_SHEEP	Fibrinogen gamma B chain	0.81	1.2	2.5951	4.0932	2.1264	5.6226	12	14	13	10	100%	100%	100%	100%
17	W5P7S6_SHEEP	Alpha-1-acid glycoprotein	0.96	1	2.2724	5.3584	1.862	5.5025	6	7	6	7	100%	100%	100%	100%
18	W5NR63_SHEEP	Protein S100	0.94	1.1	2.2985	2.0721	0.2178	4.5134	5	5	1	5	100%	100%	99%	100%
19	W5PHP7_SHEEP	Serpin A3-7	1	1	2.4956	3.2757	1.3435	4.4344	13	13	10	13	100%	100%	100%	100%
20	W5PZI1_SHEEP	Clusterin	0.81	1.1	3.25	1.9984	2.21	3.5337	16	14	16	12	100%	100%	100%	100%
21	W5PTH1_SHEEP	Vitamin D binding protein	0.33	0.7	4.5922	3.2222	2.4595	3.3034	19	14	16	13	100%	100%	100%	100%
22	A0A0M4KDI9_SHEEP	Adiponectin	0.91	1.1	1.6696	2.1844	0.8882	3.2786	4	5	3	4	100%	100%	100%	100%
23	W5P4S0_SHEEP	Ceruloplasmin	0.84	0.9	2.6374	1.4654	0.8303	2.7546	32	24	20	23	100%	100%	100%	100%
24	W5P8F9_SHEEP	Short palate, lung and nasal epithelium carcinoma associated protein	0.19	1.3	2.4279	1.8269	2.7858	2.672	5	6	5	3	100%	100%	100%	100%
25	W5P2V0_SHEEP	Ezrin	1	1	0.5322	2.3851	0.2497	2.6697	8	21	5	16	100%	100%	100%	100%
26	W5NXW9_SHEEP	Ig mu chain IgM	0.29	1.4	1.3159	1.8705	1.8856	2.584	9	10	10	8	100%	100%	100%	100%
27	W5PE01_SHEEP	Transgelin	0.96	1	1.4989	1.67	0.7534	2.3362	7	8	5	6	100%	100%	100%	100%
28	F2YQ13_SHEEP	Gelsolin isoform b	0.39	0.7	4.0587	2.1602	1.7283	2.3296	25	19	17	12	100%	100%	100%	100%
29	W5QH56_SHEEP	Alpha-2-HS-glycoprotein	0.14	0.6	3.3344	2.7124	1.5857	2.2621	12	9	9	7	100%	100%	100%	100%
30	K4P1S5_SHEEP	Peptidyl-prolyl cis-trans isomerase	0.84	0.9	1.1345	1.9236	0.3753	2.2277	4	6	2	4	100%	100%	100%	100%
31	W5P5W9_SHEEP	Triosephosphate isomerase	0.92	0.9	1.8734	1.0094	0.4642	2.1987	5	4	3	4	100%	100%	100%	100%
32	W5Q3E0_SHEEP	Thy-1 membrane glycoprotein	0.62	2	0.7515	0.407	0.1672	2.1902	2	2	1	2	100%	100%	99%	100%
33	A4ZVY6_SHEEP	Beta-2-microglobulin	0.73	0.9	1.6696	2.1844	1.3681	2.1286	4	4	4	3	100%	100%	100%	100%
34	W5PIG7_SHEEP	Alpha enolase	0.71	1.5	0.7806	0.7037	0.1966	2.0809	8	7	3	9	100%	100%	100%	100%
35	W5NXL7_SHEEP	Immunoglobulin lambda 2b light chain	0.9	1	2.523	1.3072	1.5874	2.0516	2	3	3	3	100%	100%	100%	100%
36	W5NRG7_SHEEP	Inter-alpha trypsin inhibitor heavy chain H4	0.99	1	1.8258	1.164	1.058	1.95	26	19	20	18	100%	100%	100%	100%
37	W5P1X9_SHEEP	Fructose-bisphosphate aldolase	0.43	0.6	1.8873	1.4791	0.4507	1.6692	10	9	5	7	100%	100%	100%	100%
38	W5QAB1_SHEEP	Hemopexin	0.6	1.3	1.092	0.7458	0.7826	1.6245	9	8	9	8	100%	100%	100%	100%
39	L0CSP6_SHEEP	Peroxiredoxin-5	0.56	0.6	1.1851	1.499	0.05	1.5601	3	4	0	3	100%	100%	0%	100%
40	W5QH54_SHEEP	Fetuin B	0.99	1	1.2659	1.1412	0.8902	1.5111	8	7	6	7	100%	100%	100%	100%
41	1433G_SHEEP	14-3-3 protein gamma	0.49	0.9	1.571	1.4162	1.2872	1.4873	2	2	2	1	100%	100%	100%	98%
42	W5PPQ8_SHEEP	Immunoglobulin J chain	0.78	1.3	0.4515	1.0055	0.37	1.4758	2	2	2	2	100%	100%	100%	100%
43	W5PPT6_SHEEP	Tubulin, beta 5	0.51	0.6	1.1654	1.5143	0.0589	1.475	11	13	1	8	100%	100%	99%	100%
44	THIO_SHEEP	Thioredoxin	0.39	0.5	1.3121	1.8643	0.2617	1.4628	2	3	1	2	100%	100%	83%	100%
45	W5PJZ1_SHEEP	Plasma protease C1 inhibitor	0.92	1.1	1.3541	0.6582	0.688	1.4336	10	8	7	8	100%	100%	100%	100%
46	W5P303_SHEEP	14-3-3 protein beta/alpha	0.75	1.1	1.1247	1.0139	0.9216	1.395	5	5	5	4	100%	100%	100%	100%
47	B6UV62_SHEEP	Serpin F1	0.15	0.6	2.3794	1.7106	0.9278	1.3671	14	10	9	7	100%	100%	100%	100%
48	W5PRP1_SHEEP	Na(+)/H(+) exchange regulatory cofactor NHE-RF1	0.89	1.2	0.2979	0.914	0.0742	1.3517	3	7	1	5	100%	100%	100%	100%
49	W5NY46_SHEEP	Serum paraoxonase/arylesterase	0.54	0.8	2.406	1.9089	2.2301	1.3421	11	9	10	5	100%	100%	100%	100%
50	W5NUX8_SHEEP	Complement C4	0.7	0.8	1.9644	0.8522	0.9511	1.3353	42	28	31	23	100%	100%	100%	100%
51	W5NQP5_SHEEP	Superoxide dismutase [Cu-Zn]	0.45	0.6	2.2412	1.0662	0.7492	1.3344	6	4	5	3	100%	100%	100%	100%
52	W5P824_SHEEP	Actin related protein 2/3 complex subunit 4?	0.42	1.8	0.678	0.3703	0.5555	1.3313	3	2	3	3	100%	100%	100%	100%
53	W5P4R1_SHEEP	Moesin	0.66	1.6	0.2515	0.6473	0.2061	1.2601	2	5	2	6	100%	100%	100%	100%
54	W5P3B5_SHEEP	14-3-3 protein zeta/delta	0.53	0.8	1.1039	1.8628	1.1346	1.255	5	9	7	4	100%	100%	100%	100%
55	W5P1J8_SHEEP	Amine oxidase	0.71	1.1	1.1859	0.7	0.8988	1.2479	13	11	11	10	100%	100%	100%	100%
56	W5NYA7_SHEEP	Phosphatidylethanolamine binding protein 1	0.45	0.5	2.1347	0.8239	0.1432	1.228	4	3	1	3	100%	100%	72%	100%
57	D7R7V6_SHEEP	Glyceraldehyde-3-phosphate dehydrogenase	0.42	0.5	0.9648	1.7167	0.173	1.2132	7	9	2	4	100%	100%	100%	100%
58	1433E_SHEEP	14-3-3 protein epsilon	0.67	1.2	0.6003	0.9279	0.657	1.1788	2	4	3	3	100%	100%	100%	100%
59	W5QHZ8_SHEEP	Ig kappa chain	0.52	1.5	0.594	0.5355	0.4867	1.1663	2	2	2	2	100%	100%	100%	100%

60	Q29439_SHEEP	Complement component C4	*0.018	0.6	2.2126	1.9947	1.2602	1.1557	3	3	3	1	100%	100%	100%	100%
61	W5NPI5_SHEEP	Immunoglobulin V lambda chain	0.86	1.1	0.5436	0.8311	0.4455	1.0675	2	2	2	2	100%	100%	100%	100%
62	K4P494_SHEEP	Cystatin C	0.13	1.8	0.5173	0.4663	0.715	1.0158	2	2	2	2	100%	100%	100%	100%
63	ANGT_SHEEP	Angiotensinogen	0.43	0.5	2.6659	0.8763	0.7965	0.9829	12	10	10	6	100%	100%	100%	100%
64	W5PW21_SHEEP	Inter-alpha trypsin inhibitor heavy chain H2	0.73	0.9	1.2185	0.6291	0.6189	0.9546	17	11	12	10	100%	100%	100%	100%
65	W5NTW3_SHEEP	Inter-alpha trypsin inhibitor heavy chain H1	0.23	0.7	1.2165	0.9619	0.6035	0.9032	16	14	12	9	100%	100%	100%	100%
66	W5NS06_SHEEP	Annexin	0.7	1.4	0.3168	0.4004	0.1647	0.8721	3	4	2	4	100%	100%	100%	100%
67	W5P610_SHEEP	Chloride intracellular channel protein	0.59	1.6	0.2754	0.3979	0.2256	0.8667	2	3	2	3	100%	100%	100%	100%
68	W5PHP8_SHEEP	Leucine rich alpha 2 glycoprotein	0.83	1.1	0.5539	0.4993	0.3458	0.8288	5	5	4	4	100%	100%	100%	100%
69	W5PWA8_SHEEP	Heat shock protein beta 1	0.85	0.8	0.4134	0.6155	0.05	0.8118	2	3	0	2	100%	100%	0%	100%
70	D0VWZ0_SHEEP	Tubulin, alpha chain	0.56	0.7	0.7426	0.6694	0.1211	0.8117	8	7	2	5	100%	100%	100%	100%
71	W5P101_SHEEP	Alpha 1B glycoprotein	0.32	1.5	0.4928	0.3569	0.4889	0.7774	5	5	5	5	100%	100%	100%	100%
72	W5QIK8_SHEEP	Selenium binding protein 1	0.23	0.5	0.9414	0.9699	0.2473	0.7678	8	10	4	5	100%	100%	100%	100%
73	W5NY95_SHEEP	Complement factor B	0.67	0.8	1.3788	0.4854	0.6501	0.7646	26	14	20	13	100%	100%	100%	100%
74	W5QDG7_SHEEP	Fibronectin	0.13	2.7	0.2448	0.1921	0.4524	0.7477	16	14	27	21	100%	100%	100%	100%
75	W5PXC8_SHEEP	Alpha 2 antiplasmin	0.95	1	0.6643	0.5989	0.5443	0.7336	7	6	7	5	100%	100%	100%	100%
76	W5PDE5_SHEEP	C4b binding protein alpha chain	0.39	2.2	0.8376	0.05	1.2291	0.6945	4	0	5	2	100%	0%	100%	100%
77	W5NZJ1_SHEEP	Sulfotransferase	0.81	0.8	0.4939	0.4453	0.086	0.6885	4	4	1	3	100%	100%	100%	100%
78	W5NRR7_SHEEP	Thyroxine-binding globulin	0.25	0.5	1.043	0.6932	0.269	0.6447	10	8	4	3	100%	100%	100%	100%
79	W5PUH4_SHEEP	GST pi	0.93	0.9	0.532	0.2958	0.1246	0.6442	3	2	1	2	100%	100%	98%	100%
80	W5PZM9_SHEEP	Annexin	0.91	0.9	0.8898	0.085	0.2553	0.6119	7	1	3	3	100%	100%	100%	100%
81	W5PD71_SHEEP	C-reactive protein	0.97	1	0.9683	0.6466	1.03	0.6066	5	4	5	2	100%	100%	100%	100%
82	W5P5I0_SHEEP	Complement factor I	0.68	1.1	0.4363	0.3933	0.3575	0.5784	6	7	6	5	100%	100%	100%	100%
83	W5NUE3_SHEEP	Peroxiredoxin 1	0.97	1	0.2866	0.7975	0.5397	0.5627	2	4	4	2	100%	100%	100%	100%
84	W5P6F4_SHEEP	Complement C5	0.42	0.8	0.684	0.4809	0.3686	0.5349	26	20	20	12	100%	100%	100%	100%
85	W5Q961_SHEEP	Apolipoprotein F	0.76	1.1	0.4354	0.3925	0.3567	0.5338	3	3	3	2	100%	100%	100%	100%
86	W5PDQ9_SHEEP	Complement C1g subcomponent subunit C	0.52	1.5	0.2707	0.244	0.2218	0.5315	2	2	2	2	100%	100%	100%	100%
87	W5QH50_SHEEP	Histidine rich glycoprotein	0.6	0.7	1.0409	0.384	0.4969	0.5229	10	6	7	4	100%	100%	100%	100%
88	W5PTU7_SHEEP	Carbonic anhydrase 2	0.28	0.5	0.794	0.5362	0.1008	0.5139	5	4	1	2	100%	100%	98%	100%
89	W5PK26_SHEEP	LIM and SH3 domain protein 1	0.59	0.6	0.2596	0.7088	0.05	0.5097	2	4	0	2	100%	100%	0%	100%
90	W5P671_SHEEP	N(G),N(G)-dimethylarginine dimethylaminohydrolase 2	0.9	1.1	0.2585	0.233	0.05	0.5076	2	2	0	2	100%	100%	0%	100%
91	W5NYH2_SHEEP	Nucleoside diphosphate kinase	0.96	1	0.2533	0.3643	0.0977	0.4975	2	2	1	2	100%	100%	81%	100%
92	EF1D_SHEEP	Elongation factor 1-delta	0.94	0.9	0.3973	0.2248	0.0963	0.4896	3	2	1	2	100%	100%	100%	100%
93	W5P8E9_SHEEP	CD109 antigen	0.87	0.9	0.4471	0.3203	0.2437	0.4755	14	13	12	10	100%	100%	100%	100%
94	W5NYA1_SHEEP	Complement C2	0.79	1.2	0.6826	0.1244	0.5004	0.4728	7	2	8	4	100%	100%	100%	100%
95	W5NSH2_SHEEP	Inter alpha trypsin inhibitor heavy chain H3	0.3	0.7	0.6967	0.4737	0.387	0.4598	13	10	9	6	100%	100%	100%	100%
96	W5PD15_SHEEP	Elongation factor 1-alpha	0.89	0.9	0.2301	0.4646	0.1885	0.4518	3	5	3	3	100%	100%	100%	100%
97	W5NUT0_SHEEP	polymerase 1 and transcript release factor	0.94	0.9	0.364	0.2069	0.089	0.4506	3	2	1	2	100%	100%	97%	100%
98	W5QH46_SHEEP	Kininogen 2	1	1	0.3541	0.3192	0.2352	0.4389	6	5	4	4	100%	100%	100%	100%
99	W5PXI3_SHEEP	Afamin	0.22	0.9	0.4923	0.4438	0.4034	0.4335	8	7	7	4	100%	100%	100%	100%
100	W5Q0L2_SHEEP	Plasma serine protease inhibitor	0.34	0.7	0.4891	0.4409	0.1798	0.4309	6	6	3	2	100%	100%	100%	100%
101	C8BKD1_SHEEP	Prothrombin	0.061	0.8	0.565	0.5093	0.4003	0.4305	9	8	7	4	100%	100%	100%	100%
102	W5PTE9_SHEEP	Collagen alpha 1(VI)	0.18	2.5	0.1757	0.0918	0.2427	0.4213	4	3	7	5	100%	100%	100%	100%
103	W5QFQ0_SHEEP	Malate dehydrogenase	0.8	1.1	0.2076	0.2954	0.1701	0.4077	2	2	2	2	100%	100%	100%	100%
104	ANXA2_SHEEP	Annexin A2	0.094	0.4	0.8728	0.641	0.1596	0.3825	7	5	2	2	100%	100%	100%	100%
105	W5Q2E1_SHEEP	Lumican	0.19	0.4	0.5597	0.5045	0.0757	0.3802	5	4	1	2	100%	100%	100%	100%
106	W5QJA2_SHEEP	Monocyte differentiation antigen CD14 precursor	0.19	1.7	0.1913	0.1724	0.2464	0.3756	2	2	3	2	100%	100%	100%	100%
107	W5NRF4_SHEEP	Amine oxidase	0.95	1	0.3371	0.2356	0.2141	0.3731	2	2	1	1	100%	100%	98%	85%
108	W5P323_SHEEP	Glucose-6-phosphate isomerase	0.58	1.6	0.1165	0.1622	0.0954	0.3532	2	3	2	2	100%	100%	100%	100%
109	D8X187_SHEEP	Serpin peptidase inhibitor clade B ovalbumin member 1	0.73	0.8	0.2779	0.2505	0.0696	0.3482	3	3	1	2	100%	100%	100%	100%
110	W5QA64_SHEEP	HP-20 homolog	0.65	1.2	0.3617	0.15	0.2964	0.3268	2	1	2	1	100%	100%	100%	100%
111	W5P3R3_SHEEP	Plasminogen	0.76	0.8	0.3581	0.1896	0.1351	0.3238	8	5	4	3	100%	100%	100%	100%
112	W5PN97_SHEEP	Tetranectin	0.34	0.4	1.5224	0.5262	0.4783	0.3234	4	3	3	1	100%	100%	100%	98%
113	W5NQW4_SHEEP	Alpha 1 macroglobulin	0.83	0.8	1.0608	0.1885	0.6976	0.3122	30	9	26	6	100%	100%	100%	100%
114	W5Q4Z3_SHEEP	Insulin like growth factor binding protein complex acid labile subunit	0.88	0.9	0.3434	0.143	0.13	0.3115	4	2	2	2	100%	100%	100%	100%
115	W5QAQ1_SHEEP	Leucine rich repeat neuronal protein 4	0.72	0.8	0.2723	0.1913	0.05	0.3046	5	4	1	3	100%	100%	99%	100%
116	W5PTR5_SHEEP	78 kDa glucose regulated protein	0.49	2.5	0.05	0.0905	0.05	0.3032	0	2	1	3	85%	100%	100%	100%
117	W5PFJ0_SHEEP	Vinculin	0.3	0.6	0.4	0.2781	0.1497	0.2818	10	8	5	4	100%	100%	100%	100%
118	W5P3J3_SHEEP	Complement C1s subcomponent	0.39	2.1	0.1432	0.05	0.1173	0.2811	2	1	3	3	100%	97%	100%	100%
119	W5PGT6_SHEEP	Complement component C6	0.75	0.9	0.4431	0.2364	0.3241	0.279	11	7	9	4	100%	100%	100%	100%
120	W5Q0V2_SHEEP	Biotindase	0.079	3.5	0.0666	0.06	0.1754	0.2707	1	1	3	2	100%	96%	100%	100%
121	W5PNW7_SHEEP	Vimentin	0.39	0.6	0.2948	0.2657	0.05	0.2701	4	3	0	1	100%	100%	98%	98%

122	W5PVL4_SHEEP	Mannose binding protein	0.45	1.5	0.2919	0.2631	0.551	0.2676	2	2	3	1	100%	100%	100%	100%
123	W5QA34_SHEEP	Vascular cell adhesion protein-1	0.15	3	0.0885	0.05	0.1517	0.2666	2	0	4	3	100%	0%	100%	100%
124	W5PJ98_SHEEP	F actin capping protein	0.24	0.5	0.2892	0.4192	0.1107	0.2653	2	3	1	1	100%	100%	100%	99%
125	W5PB46_SHEEP	Phopholipid transfer protein	0.75	1.3	0.2798	0.0571	0.1663	0.2572	4	1	2	2	100%	82%	100%	100%
126	W5PUC1_SHEEP	Carbonic anhydrase 3	0.3	0.4	0.4182	0.2359	0.05	0.2415	3	2	0	1	100%	100%	61%	100%
127	W5PK03_SHEEP	Periostin	*0.041	5.8	0.05	0.05	0.3415	0.2407	0	0	9	3	88%	0%	100%	100%
128	W5QI29_SHEEP	Extracellular matrix protein 1	0.99	1	0.1214	0.1692	0.05	0.2384	2	3	1	2	100%	100%	100%	100%
129	W5PDJ4_SHEEP	Platelet activating factor acetylhydrolase 1B subunit gamma isoform	0.4	0.6	0.2533	0.2284	0.05	0.2342	2	2	0	1	100%	100%	0%	100%
130	W5PH81_SHEEP	Complement component C7	*0.011	0.6	0.3483	0.3605	0.2055	0.232	8	8	6	3	100%	100%	100%	100%
131	W5P880_SHEEP	Proteoglycan 4	0.3	1.9	0.1463	0.05	0.1459	0.2265	5	1	6	4	100%	100%	100%	100%
132	W5NR02_SHEEP	Estrogen response element binding protein	0.4	0.7	0.2427	0.2188	0.0938	0.2248	2	2	1	1	100%	100%	98%	98%
133	W5P707_SHEEP	Alpha actinin 4	0.28	0.3	0.8642	0.3276	0.1877	0.2129	17	9	6	3	100%	100%	100%	100%
134	W5PBY0_SHEEP	C4b binding protein alpha chain	0.41	1.4	0.1061	0.1473	0.1339	0.2083	2	3	3	2	100%	100%	100%	100%
135	W5Q9A2_SHEEP	Zinc alpha glycoprotein	0.4	0.7	0.3382	0.1929	0.1753	0.1995	3	2	2	1	100%	100%	100%	98%
136	W5PI61_SHEEP	Apolipoprotein E	0.58	0.6	1.1507	0.2975	0.6337	0.1951	8	3	6	1	100%	100%	100%	96%
137	W5PGE9_SHEEP	Ig alpha heavy chain IgA	0.77	0.9	0.3147	0.1801	0.2579	0.1868	3	2	3	1	100%	100%	100%	100%
138	W5Q268_SHEEP	Beta 2 glycoprotein 1	0.4	0.7	0.3106	0.1778	0.1616	0.1846	3	2	1	1	100%	100%	100%	98%
139	W5PX38_SHEEP	Mesothelin like	0.46	0.9	0.1773	0.1599	0.1453	0.1667	2	2	2	1	100%	100%	100%	100%
140	W5PFF9_SHEEP	Complement factor H	0.4	1.3	0.1638	0.0958	0.1838	0.1545	2	2	2	2	100%	100%	100%	100%
141	W5Q0R1_SHEEP	Sex hormone binding globulin	0.54	0.8	0.1619	0.3164	0.2071	0.1527	2	4	3	1	100%	100%	100%	98%
142	W5PFV5_SHEEP	Puromycin sensitive aminopeptidase	0.49	0.6	0.2515	0.1068	0.0634	0.152	6	3	2	2	100%	100%	100%	100%
143	W5PN88_SHEEP	Elongation factor 2	0.91	1.1	0.1156	0.0681	0.05	0.1484	3	2	1	2	100%	100%	96%	100%
144	W5Q517_SHEEP	Procollagen C endopeptidase enhancer 1	*0.026	0.6	0.2394	0.2158	0.1259	0.1452	3	3	2	1	100%	100%	100%	90%
145	W5PE22_SHEEP	Rab GDP dissociation inhibitor beta	0.073	0.3	0.3161	0.4609	0.058	0.139	4	6	1	1	100%	100%	100%	100%
146	W5PPG3_SHEEP	4 trimethylaminobutyraldehyde dehydrogenase	0.42	0.7	0.1441	0.1299	0.05	0.1366	2	2	0	1	100%	100%	0%	94%
147	W5Q4Q3_SHEEP	Heparin cofactor 2 precursor	0.24	0.6	0.2887	0.1887	0.1715	0.1281	4	3	3	1	100%	100%	100%	100%
148	W5QI69_SHEEP	Retinal dehydrogenase 2 isoform 1	0.088	0.4	0.2013	0.25	0.05	0.1235	3	4	0	1	100%	100%	65%	93%
149	W5QC41_SHEEP	Pyruvate kinase	0.37	0.5	0.1189	0.2933	0.0974	0.1133	2	5	2	1	100%	100%	100%	98%
150	W5PID9_SHEEP	Complement component C9	*0.036	0.5	0.251	0.2915	0.1497	0.1127	4	5	3	1	100%	100%	100%	100%
151	D6PZY4_SHEEP	Factor H	0.62	0.8	0.2151	0.1638	0.2045	0.1121	7	5	8	2	100%	100%	100%	100%
152	W5PUE4_SHEEP	Fibrocystin L	0.12	1.6	0.0776	0.05	0.0972	0.1054	10	7	15	7	100%	100%	100%	100%
153	W5QFP0_SHEEP	Thrombospondin 1	0.33	0.6	0.1599	0.0936	0.05	0.0993	5	4	2	2	100%	100%	100%	100%
154	W5PEI4_SHEEP	Plasma kallikrein	0.26	0.6	0.1021	0.1417	0.05	0.0977	2	3	1	1	100%	100%	100%	95%
155	W5Q3F9_SHEEP	Aldehyde oxidase	0.34	0.5	0.1801	0.0893	0.05	0.0949	6	4	0	2	100%	100%	30%	100%
156	W5Q749_SHEEP	Vitamin K dependent protein S	0.84	0.9	0.203	0.0871	0.1664	0.0926	4	2	4	1	100%	100%	100%	100%
157	W5PQK6_SHEEP	Talin 1	0.49	0.8	0.0669	0.0984	0.0548	0.0777	5	8	5	3	100%	100%	100%	100%
158	W5P5A0_SHEEP	Filamin A	0.61	0.8	0.0914	0.0581	0.05	0.0749	7	5	1	3	100%	100%	100%	100%
159	W5PT68_SHEEP	Filamin B isoform X1	0.92	1	0.0638	0.0575	0.05	0.0742	5	5	3	3	100%	100%	100%	100%
160	W5PYS8_SHEEP	Complement C3	0.41	0.6	0.1388	0.0694	0.0797	0.05	7	4	4	1	100%	100%	100%	100%
161	W5Q5Q6_SHEEP	Collagen alpha 1 (XIV)	0.098	0.3	0.2138	0.1308	0.05	0.05	10	7	0	1	100%	100%	0%	100%
162	W5Q4S0_SHEEP	Collagen alpha 1 (III)	0.42	0.6	0.1059	0.05	0.05	0.05	3	2	0	1	100%	100%	13%	77%
163	AL1A1_SHEEP	Retinal dehydrogenase 1	0.2	0.2	0.2874	0.1211	0.05	0.05	4	2	0	0	100%	100%	0%	55%
164	W5NX96_SHEEP	Attractin	0.53	0.7	0.1015	0.05	0.0616	0.05	4	2	3	0	100%	100%	100%	46%
165	W5PUT6_SHEEP	Clathrin heavy chain 1	0.42	1	0.05	0.0505	0.05	0.05	2	3	0	0	100%	100%	0%	44%
166	W5P5Q2_SHEEP	Major vault protein	0.056	0.3	0.192	0.1359	0.05	0.05	5	4	0	0	100%	100%	0%	0%
167	W5PGC5_SHEEP	Aldose 1-epimerase	*0.018	0.3	0.2004	0.1806	0.0782	0.05	2	2	1	0	100%	100%	94%	0%
168	W5PJ75_SHEEP	Spectrin alpha chain	1	1	0.05	0.05	0.05	0.05	3	3	0	0	100%	100%	0%	0%
169	W5NXJ3_SHEEP	Complement C4	0.12	0.3	0.4335	0.2791	0.1611	0.05	2	2	0	0	100%	100%	63%	0%
170	W5PEL2_SHEEP	Mimecan isoform 1	0.2	0.4	0.2222	0.3172	0.1821	0.05	2	3	2	0	100%	100%	100%	0%
171	W5PD62_SHEEP	Carboxypeptidase B2	*0.05	0.3	0.1525	0.2142	0.05	0.05	2	3	0	0	100%	100%	34%	0%
172	W5P812_SHEEP	Protein AMBP	0.28	0.6	0.1919	0.173	0.1572	0.05	2	2	2	0	100%	100%	100%	0%
173	W5P8I3_SHEEP	Ribonucelase UK114	0.26	0.3	1.0171	0.5355	0.4867	0.05	3	2	2	0	100%	100%	100%	0%
174	W5Q9B2_SHEEP	Rho GDP dissociation inibitor 1	0.091	0.1	0.541	0.3004	0.05	0.05	3	2	0	0	100%	100%	0%	0%
175	W5P9U4_SHEEP	Proteasome subunit alpha type	*0.043	0.2	0.2606	0.3754	0.05	0.05	2	3	0	0	100%	100%	0%	0%
176	W5P5W6_SHEEP	Protein NDRG1	*0.0057	0.3	0.167	0.1506	0.05	0.05	2	2	0	0	100%	100%	0%	0%

Supplementary Figure 1

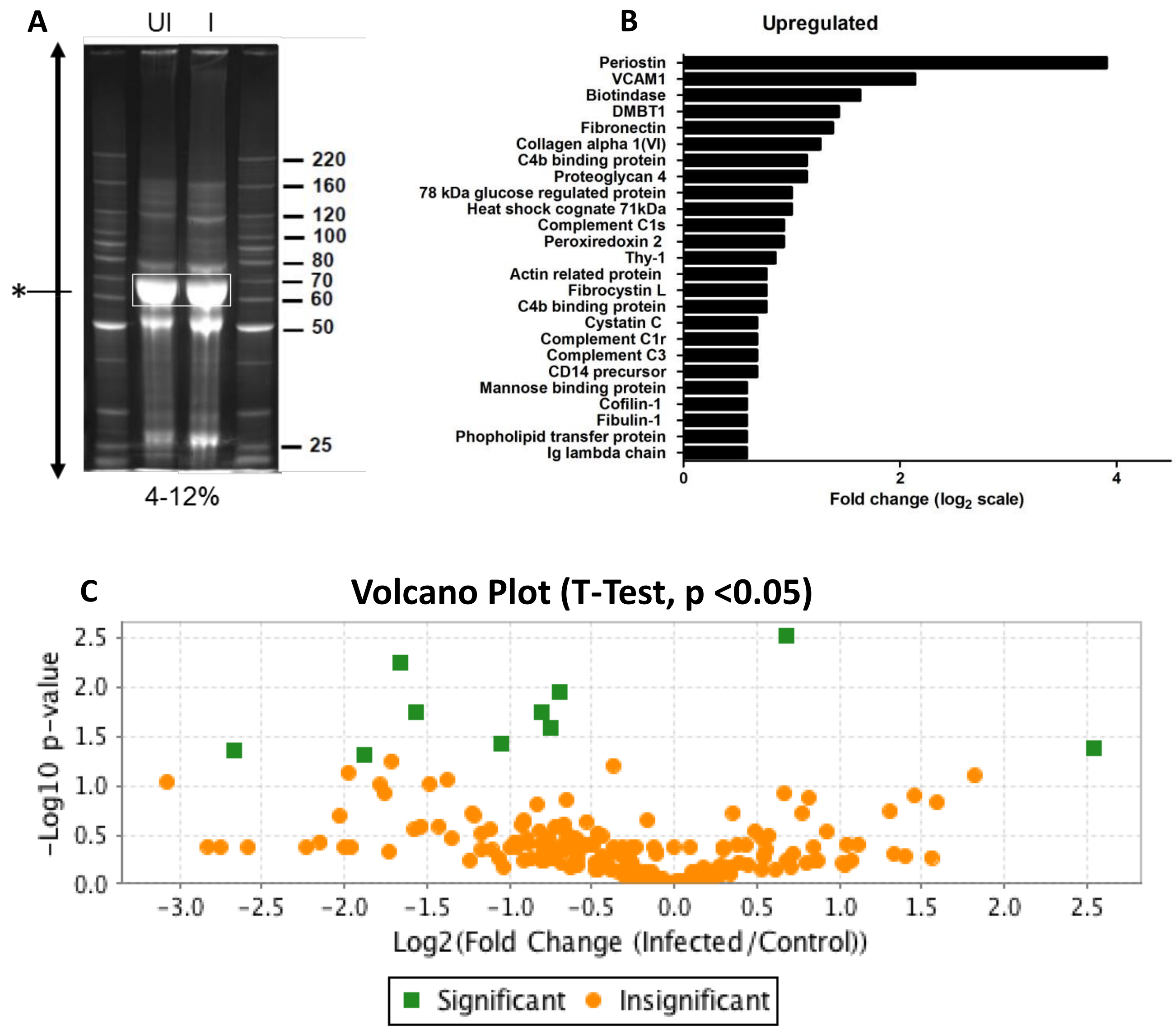

Supplement: Supplementary file 1 — Supplementary Information [file 41598_2017_3094_MOESM1_ESM.pdf]
